# Activation of Transcription Factor EB Is Associated With Adipose Tissue Lipolysis in Dairy Cows With Subclinical Ketosis

**DOI:** 10.3389/fvets.2022.816064

**Published:** 2022-02-08

**Authors:** Hao Yu, Xinxing Gao, Juan J. Loor, Qianming Jiang, Zhiyuan Fang, Xue Hao, Zhen Shi, Minghe Fan, Meng Chen, Xinwei Li, Guowen Liu, Zhe Wang, Xiaobing Li, Xiliang Du

**Affiliations:** ^1^Key Laboratory of Zoonoses Research, Ministry of Education, College of Veterinary Medicine, Jilin University, Jilin, China; ^2^Mammalian NutriPhysioGenomics, Department of Animal Sciences and Division of Nutritional Sciences, University of Illinois, Urbana, IL, United States; ^3^College of Veterinary Medicine, Yunnan Agricultural University, Kunming, China

**Keywords:** adipocytes, isoproterenol, lipolysis, transcription factor EB, autophagy-lysosomal pathway

## Abstract

Excessive lipid mobilization for adipose tissue caused by severe negative energy balance is the pathological basis for subclinical ketosis (SCK) in dairy cows. In non-ruminants, transcription factor EB (TFEB) was reported to play a role in the regulation of lipid catabolism, but its role in the control of lipolysis in the bovine is unknown. The present study aimed to determine whether the enhanced TFEB transcriptional activity contributes to lipolysis of adipose tissue in SCK cows, and to explore the possibility of establishing a therapeutic strategy by using TFEB as a target to control lipolysis. Thirty cows with similar lactation number (media*n* = 3, range = 2–4) and days in milk (media*n* = 6 d, range = 3–9) were selected into a healthy control (*n* = 15) and SCK (*n* = 15) group, and used for subcutaneous adipose tissue biopsies and blood sampling. Adipocytes from healthy Holstein calves were used as a model for *in vitro* studies involving treatment with 10 μ*M* isoproterenol (ISO) for 0, 1, 2 and 3 h, 250 n*M* of the TFEB activator Torin1 for 3 h, or used for transfection with TFEB small interfering RNA for 48 h followed by treatment with 10 μ*M* ISO for 3 h. Compared with healthy cows, adipose tissue in SCK cows showed increased lipolysis accompanied by enhanced TFEB transcriptional activity. *In vitro*, ISO and Torin1 treatment increased lipolysis and enhanced TFEB transcriptional activity in calf adipocytes. However, knockdown of *TFEB* attenuated ISO-induced lipolysis in adipocytes. Overall, these findings indicated that enhanced transcriptional activity of TFEB may contribute to lipolysis of adipose tissue in dairy cows with SCK. The regulation of TFEB activity may be an effective therapeutic strategy for controlling overt lipolysis in ketotic cows.

## Introduction

During the early post-partal period, increased nutrient requirements for lactogenesis and decreased dry matter intake (DMI) lead to the occurrence of negative energy balance [NEB; (1–3)]. Subsequently, white adipose tissue (WAT), the main storage site of triglyceride, is rapidly mobilized to produce a large amount of free fatty acids (FFA), which are important substrates for energy production and milk fat synthesis (4–6). Excessive FFA absorbed by the liver are incompletely oxidized to ketone bodies [β-hydroxybutyrate (BHB), acetoacetate and acetone], resulting in the occurrence of ketosis (7–9).

The transcription factor EB (TFEB), a basic helix-loop-helix leucine zipper transcription factor belonging to the microphthalmia/transcription factor E (MiT/TFE) family, plays an essential role in lipid homeostasis (10, 11). Under nutrient-rich conditions, TFEB is phosphorylated by multiple kinases, and phosphorylated TFEB is then retained in the cytoplasm by interacting with tyrosine 3-monooxygenase/tryptophan 5-monooxygenase activation protein [YWHA/14-3-3; (12–14)]. Under nutrient-deficient conditions, TFEB is dephosphorylated by calcineurin, dissociates from YWHA/14-3-3 proteins and translocates to the nucleus, in which it initiates the transcription of target genes involved in lipid catabolism and the autophagy-lysosomal pathway (15, 16). It has been reported that knockout of hepatic *TFEB* decreased lipolysis and increased lipid synthesis in the liver of mice fed with high fat diet [HFD; (10)]. Furthermore, adipocyte-specific *TFEB* overexpression enhanced lipolysis in WAT of HFD mice (17). Although dairy cows with ketosis are characterized by sustained lipolysis, the transcriptional activity of TFEB and its role in the regulation of lipolysis has not been evaluated.

At least in non-ruminants, activated TFEB upregulates the abundance of genes required for autophagosome formation and lysosome biogenesis (13, 18, 19). Thus, upregulation of autophagy-related gene 5 (ATG5), ATG7 and microtubule associated protein 1 light chain 3 (LC3)-II, which are essential for autophagosome formation, in WAT of ketotic cows suggested that TFEB may be activated to upregulate the expression of these molecules (20). In addition, negative nutrient balance is an effective trigger of TFEB nuclear translocation in *C. elegans* and mice (10, 21, 22). Considering that dairy cows with ketosis are in a state of NEB and sustained WAT mobilization, we hypothesized that enhanced transcriptional activity of TFEB may partly contribute to lipolysis during this physiological state. The objectives of this study were to determine whether the enhanced TFEB transcriptional activity contributes to lipolysis of adipose tissue in SCK cows, and to explore the possibility of establishing a therapeutic strategy by using TFEB as a target to control lipolysis.

## Materials and Methods

### Animals

The animal use protocol was approved by the Ethics Committee on the Use and Care of Animals at Jilin University (Changchun, China, 2018; clinical trial 201804010). Dairy cows were selected from a commercial dairy farm located in Changchun, Jilin Province, China. All cows in this study with a similar number of lactations (median = 3, range = 2–4) and DIM (median = 6 d, range = 3–9) were offered a TMR [reported in our previous publication; (23)] for *ad libitum* intake at 0730 and 1,330 h daily. Concentrations of BHB in blood of those cows was measured for 3 consecutive days to ascertain ketosis status. Fifteen cows with blood BHB concentration below 1.2 m*M* were selected as the healthy control group and 15 cows with blood BHB concentration 1.2 to 3 m*M* were classified as subclinical ketosis (24, 25). These cows were kept separately in a tie-stall barn. The basic description of the cows used was reported in our previous manuscript (23).

### Sample Collection

Subcutaneous WAT (approximately 3 g) biopsies from each of the fifteen cows were harvested as previously described (26). Briefly, the biopsy site, a 5 × 5 cm area of skin at the tailhead, was shaved, then sanitized with iodine scrub and 75% alcohol, and finally anesthetized with 2% lidocaine HCl (Sigma-Aldrich Co., St. Louis, MO, USA). A 1.5–2.5-cm scalpel incision through the skin and subcutaneous tissue is made by aseptic techniques. Adipose tissue was clamped with tweezers and snipped with scissors. After the samples were rinsed with saline, a portion was rapidly frozen in liquid nitrogen and stored at −80°C until analysis, while the rest was fixed in 4% formalin. The tissue specimens were then embedded in paraffin blocks using routine procedures, followed by hematoxylin and eosin (HE) staining and histopathological examination for morphological changes under a microscope.

### Isolation of Primary Preadipocytes

The isolation of primary preadipocytes was performed according to published procedures with minor modification (27). Briefly, adipose tissue was surgically obtained from the peritoneal omentum and mesentery of 5 healthy Holstein calves (1 d old, female, 30–40 kg, fasting) under sterile conditions. Subsequently, adipose tissue was rinsed in sterile phosphate buffered saline (PBS) containing penicillin (2,500 U/mL) and streptomycin (2,500 μg/mL) to remove adherent blood. The fascia and blood vessels visible in the tissue were peeled away, and the resulting adipose tissue was cut into small pieces of ~1 mm^3^, digested using 50 mL of Dulbecco's modified Eagle's medium (DMEM)/F12 (SH30023.01; HyClone, Logan, Utah, USA) digestion solution containing collagenase type I (1 mg/mL; C0130; Sigma-Aldrich) at 37°C and incubated in a shaking water bath for 1.5 h. The mixture was removed through 80- and 40-μm cell filters in sequence and the filtrate was centrifuged at 175 × g for 10 min at room temperature. The residual erythrocytes were removed by adding ACK lysis buffer (C3702; Beyotime Institute of Biotechnology, Jiangsu, China) into the resulting cell pellet and centrifuging at 175 × g for 10 min at room temperature. The supernatant was discarded, and the resulting cell pellet was resuspended with basal culture medium (BCM), which was DMEM/F12 with 10% fetal bovine serum (SH30084.03; HyClone) and 1% penicillin-streptomycin (Sv30010; HyClone). The cell suspension (1 × 10^4^ cells/mL) was seeded in a cell culture flask. Preadipocytes were then incubated at 37°C in 5% CO_2_ and saturated humidity in a cell incubator for 24 h and the medium was replaced to remove non-adherent cells and tissue residues. Then, BCM was replaced every other day until the next experiment.

### Cell Culture

Primary preadipocytes were seeded in 6-well cell culture plates (Corning Costar Corp., Cambridge, MA, USA) and cultured in BCM. After cells were ~70% confluent, the BCM was replaced by freshly-prepared differentiation culture medium 1 (DCM1) adding 0.5 mM 3-Isobutyl-1-methylxanthin (IBMX; I-7018; Sigma-Aldrich), 1 μ*M* dexamethasone (D-4902; Sigma-Aldrich) and 1 μg/mL insulin (I-5500; Sigma-Aldrich) in BCM to induce preadipocytes differentiation. After 2 days, DCM1 was replaced with differentiation culture medium 2 (DCM2), which contained a final concentration of 1 μg/mL insulin in BCM, to maintain the differentiation state. Fresh DCM2 was replaced every other day for about 10 days until visible lipid droplets appeared in the cell, indicating that cells had completed differentiation. After differentiation, the number of mature adipocytes was 4.0 × 10^5^ per 6-well plate.

### Cell Treatment

To stimulate lipolysis, mature adipocytes were incubated with DMEM/F12 containing 10 μ*M* isoproterenol (ISO; S2566; Selleck Chemicals, Houston, TX, USA) for 0, 1, 2 and 3 h as previously reported (28). To activate TFEB, mature adipocytes were incubated with DMEM/F12 containing 250 n*M* Torin1 (SC0245; Beyotime Institute of Biotechnology, Jiangsu, China) for 3 h (29, 30). To silence *TFEB*, mature adipocytes were transfected with 100 pmol small interfering RNA (siRNA) of TFEB or negative control with lipofectamine 2000 (11668019; Invitrogen, Carlsbad, CA) in 2 mL DMEM/F12 for 48 h. The specific siRNA for bovine *TFEB* was designed based on the bovine *TFEB* mRNA sequence (NM_001205666.1) and synthesized by Sangon Biotech (Shanghai, China). The siTFEB sequences were as follows: the sense 5′-UGUCCAGCAGUCACCUGAAUGUGUATT-3′ and antisense 5′-ACAGGUCGUCAGUGGACUUACACAUTT-3′. The siControl sequences were as follows: the sense 5′-UUCUCCGAACGUGUCACGUTT-3′ and antisense 5′-ACGUGACACGUUCGGAGAATT-3′. All experiments were repeated in five calves and at least three technical replicates were performed in each calf.

### Oil Red O Staining

After the above treatment, adipocytes were stained with Oil Red O to evaluate intracellular lipid accumulation. The cells were washed three times in PBS, then fixed with 4% paraformaldehyde for 15 min. After washing three times with PBS, the cells were washed in 60% (vol/vol) isopropanol for 2 min, stained with freshly prepared 0.5% (wt/vol) oil red O solution for 15 min, and washed with 60% isopropanol, followed by washing three times with PBS. After counter-stained with hematoxylin, the lipid droplets were observed under a microscope.

### Glycerol Content Determination

The cell-free supernatant from adipocyte cultures was collected to measure glycerol (GC) content using an enzymatic kit (E1002; Applygen Technologies, Beijing, China) following the manufacturer's instructions. A total of 50 μL supernatant was mixed with 150 μL of chromogenic liquid and incubated at 37°C for 15 min. The absorbance at 550 nm is proportional to the concentration of GC in each sample.

### Triglyceride Content Determination

Triglyceride (TG) content in adipocytes was determined using a commercial kit (E1013; Applygen Technologies Inc.) according to the manufacturer's protocols. Approximately 1 × 10^6^ cells were mixed with 0.1 mL of lysis buffer, and kept at room temperature for 10 min. Part of the supernatant was taken to determine the total protein concentrations using the bicinchoninic acid assay (BCA assay; P1511, Applygen Technologies Inc.). The remaining supernatant was heated in water bath at 70°C for 10 min, and was centrifuged at 800 × g for 5 min at room temperature. A total of 10 μL supernatant was then mixed with 190 μL of chromogenic liquid and incubated at 37°C for 15 min. The absorbance at 550 nm was proportional to the concentration of TG in each sample.

### Quantitative Real-Time PCR Analysis

Total RNA was isolated from adipose tissue and adipocytes using RNAiso Plus (9109; TaKaRa Biotechnology Co. Ltd., Dalian, China) according to the manufacturer's instructions. The RNA concentration and quality were measured using a Nanophotometer N50 Touch (Implen GmbH, Munich, Germany) and by electrophoresis (1% agarose gels). The cDNA was then reverse-transcripted from 2 μg of purified total RNA in each sample in a 40-μL reaction using a reverse transcription kit (RR047A; TaKaRa Biotechnology Co. Ltd.) according to the supplier's protocol. Relative mRNA abundance of target genes was analyzed by quantitative real-time (qRT)-PCR technology with the SYBR Green plus reagent kit (RR420A; TaKaRa Biotechnology Co. Ltd.) and a 7500 Real-Time PCR System (Applied Biosystems Inc., Waltham, MA). The reaction conditions were as follows: 95°C for 30 s, followed by 45 cycles of 95°C for 5 s and 60°C for 34 s. Relative transcription of each target gene was normalized to glyceraldehyde-3-phosphate dehydrogenase (*GAPDH*) and β-actin (*ACTB*). Gene expression was calculated with the 2^−Δ*ΔCT*^ method, where CT is the cycle threshold. For *in vivo* qRT-PCR experiments, the PCR reaction was performed in triplicate for each of the 15 cows per group. For *in vitro* qRT-PCR experiments, the PCR reaction was performed in triplicate for each of the 5 calves. Primers of target genes were designed by Primer Express software (Applied Biosystems Inc.) and sequences are summarized in Supplementary Table 1. A melting curve was analyzed to ensure the absence of primer dimers and other non-specific amplification products.

### Protein Extraction and Western Blotting Analysis

Western blotting was performed according to published procedures with minor modifications (31). Total protein was extracted from adipose tissue or adipocytes using a commercial protein extraction kit containing lysate buffers, phosphatase inhibitors, and protease inhibitors (C510003; Sangon Biotech Co. Ltd., Shanghai, China) according to the manufacturer's instructions. Nuclear and cytoplasmic protein were extracted from adipose tissue or adipocytes using a commercial kit containing nuclear and cytoplasmic extraction buffers (P1200; Applygen Technologies Inc.) according to the manufacturer's instructions. Protein concentrations were quantified using the BCA assay (P1511; Applygen Technologies Inc.). Twenty micrograms of protein from each sample were separated by 10 or 12% SDS-PAGE and electrophoretically transferred onto 0.45 μm polyvinylidene difluoride membrane. Membranes were blocked in 3% bovine albumin and Tris-buffered saline solution with 0.01% Tween-20 for 4 h at room temperature. Subsequently, blocked membranes were incubated with primary antibodies against phosphorylated (p)-TFEB (Ser142; bs-22337R; Bioss Biotechnology Co. Ltd., Beijing, China; 1:1,000), TFEB (13372-1-AP; Proteintech, Wuhan, China; 1:1000), phosphorylated hormone sensitive lipase [p-HSL (Ser563); AF2350; Affinity Biosciences Ltd., Jiangsu, China; 1:1,000], HSL (AF6403; Affinity Biosciences Ltd.; 1:1,000), adipose triacylglycerol lipase (ATGL; Ab99532; Abcam, Cambridge, MA; 1:1,000), Histone H3 (4499; Cell Signaling Technology; 1:1,000), β-actin (ACTB; Ab8226; Abcam; 1:2,000), β-tubulin (10094-1-AP; Proteintech; 1:1,000) at 4 °C overnight, followed by washing 3 times with Tris-buffered saline solution with 0.01% Tween-20. Membranes were then incubated at room temperature with horseradish peroxidase-conjugated anti-mouse or anti-rabbit secondary antibody (Boster Biological Technology Co. Ltd., Wuhan, China) for 45 min. After washing with Tris-buffered saline solution with 0.01% Tween-20 for 3 times, the immunoassay was performed using an enhanced chemiluminescence reagent (WBKLS0500; Millipore, Bedford, MA) to visualize bands on the membrane. Lastly, all bands were analyzed using Image-Pro Plus 6.0 (Media Cybernetics Inc., Warrendale, PA). In this study, phosphorylation level of TFEB was calculated as p-TFEB/total TFEB, phosphorylation level of HSL was calculated as p-HSL/total HSL, nuclear protein abundance of TFEB was normalized to Histone H3, cytoplasmic protein abundance of TFEB was normalized to β-tubulin, and the remaining target protein abundance was normalized to ACTB.

### Statistical Analysis

All data were analyzed using GraphPad Prism 5.0 (Graph Pad Software, San Diego, CA) or SPSS 19.0 software (SPSS Inc., Chicago, IL). All data were tested for normality and homoscedasticity using the Shapiro-Wilk and Levene tests, respectively. For *in vivo* studies, parametric statistical analysis was performed using the unpaired *t*-tests. For *in vitro* studies, linear and quadratic contrasts were conducted to evaluate time-dependent effects; comparisons among groups were calculated using one-way ANOVA or two-way ANOVA with Tukey's tests for data meeting homogeneity of variance or with Tamhane's T2 analysis for data of heteroscedasticity. Data are expressed as means ± standard errors of the mean. *P* < 0.05 was considered statistically significant and *P* < 0.01 was marked significant.

## Results

### Lipolysis in White Adipose Tissue

HE staining showed that the adipocytes of SCK cows were smaller (Supplementary Figure 1A) than those of healthy cows. The mRNA abundance of acetyl-CoA carboxylase 1 (*ACC1*) and diacylglycerol acyltransferase 1 (*DGAT1*) in adipose tissue of SCK cows was slight lower than healthy cows (*P* > 0.05, Supplementary Figures 1B,C). Compared with healthy cows, the ratio of p-HSL to HSL was greater (*P* < 0.01, Supplementary Figures 1E,F) in WAT of dairy cows with SCK. Furthermore, the mRNA and protein abundance of ATGL was greater in WAT of dairy cows with SCK (*P* < 0.01, Supplementary Figures 1D,E,G).

### Transcriptional Activity of Transcription Factor EB in White Adipose Tissue

**Figure 1:**
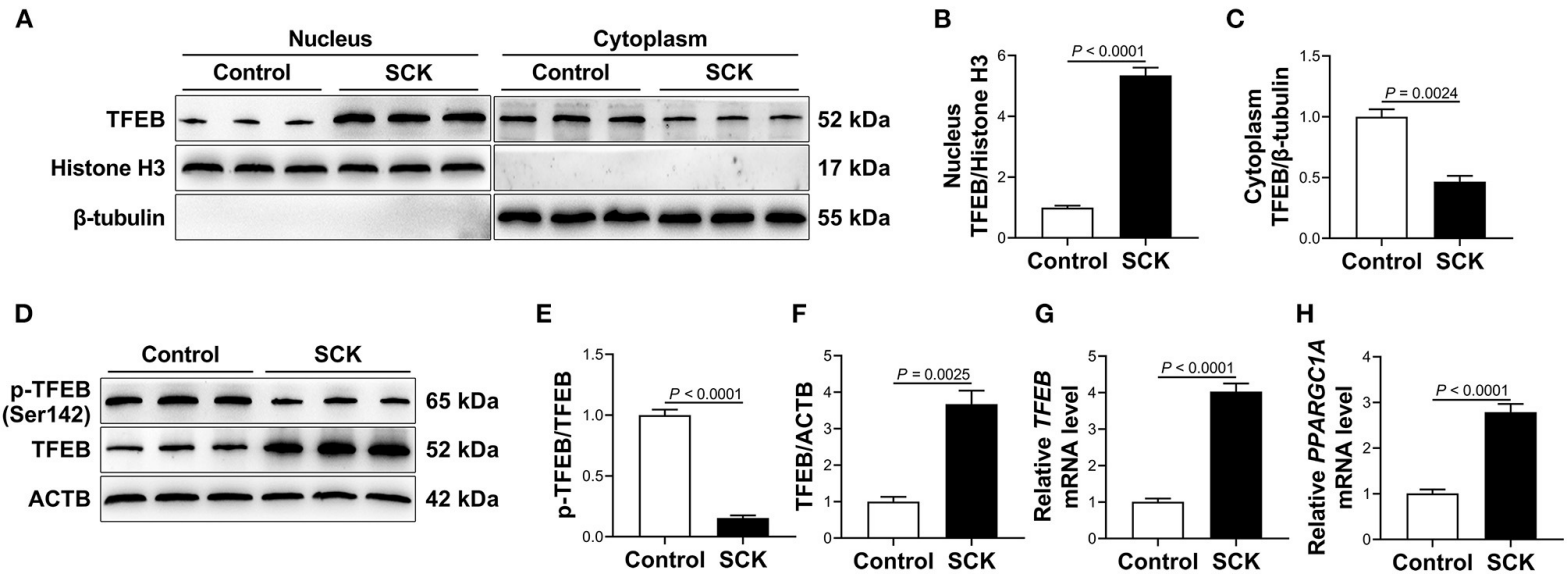
Transcriptional activity of transcription factor EB (TFEB) in white adipose tissue (WAT). **(A)** Western blot analysis of TFEB in the nucleus and cytoplasm of WAT of control cows (*n* = 15) and dairy cows with subclinical ketosis (SCK; *n* = 15). Representative blots are shown. **(B,C)** Quantification of protein abundance of nuclear and cytoplasmic TFEB, respectively. **(D)** Western blot analysis of phosphorylated TFEB (p-TFEB) and total TFEB in WAT of control cows (*n* = 15) and dairy cows with SCK (*n* = 15). Representative blots are shown. **(E,F)** Quantification of ratio of p-TFEB/TFEB and protein abundance of TFEB, respectively. **(G,H)** Relative mRNA abundance of *TFEB* and peroxisome proliferator-activated receptor gamma coactivator 1 alpha (*PPARGC1A*) in WAT of control cows (*n* = 15) and dairy cows with SCK (*n* = 15). Data were analyzed using unpaired *t*-tests and expressed as mean ± SEM.

Nuclear and total protein abundance of TFEB were greater (*P* < 0.01, Figures 1A,B,D,F), while cytoplasmic protein abundance of TFEB and ratio of p-TFEB to TFEB were lower (*P* < 0.01, Figures 1A,C–E) in WAT of dairy cows with SCK. In addition, mRNA abundance of *TFEB* and its downstream target gene peroxisome proliferator-activated receptor gamma coactivator 1 alpha (*PPARGC1A*) was greater (*P* < 0.01, Figures 1G,H) in WAT of dairy cows with SCK.

### Effects of Isoproterenol and Torin1 on Transcriptional Activity of Transcription Factor EB

**Figure 2:**
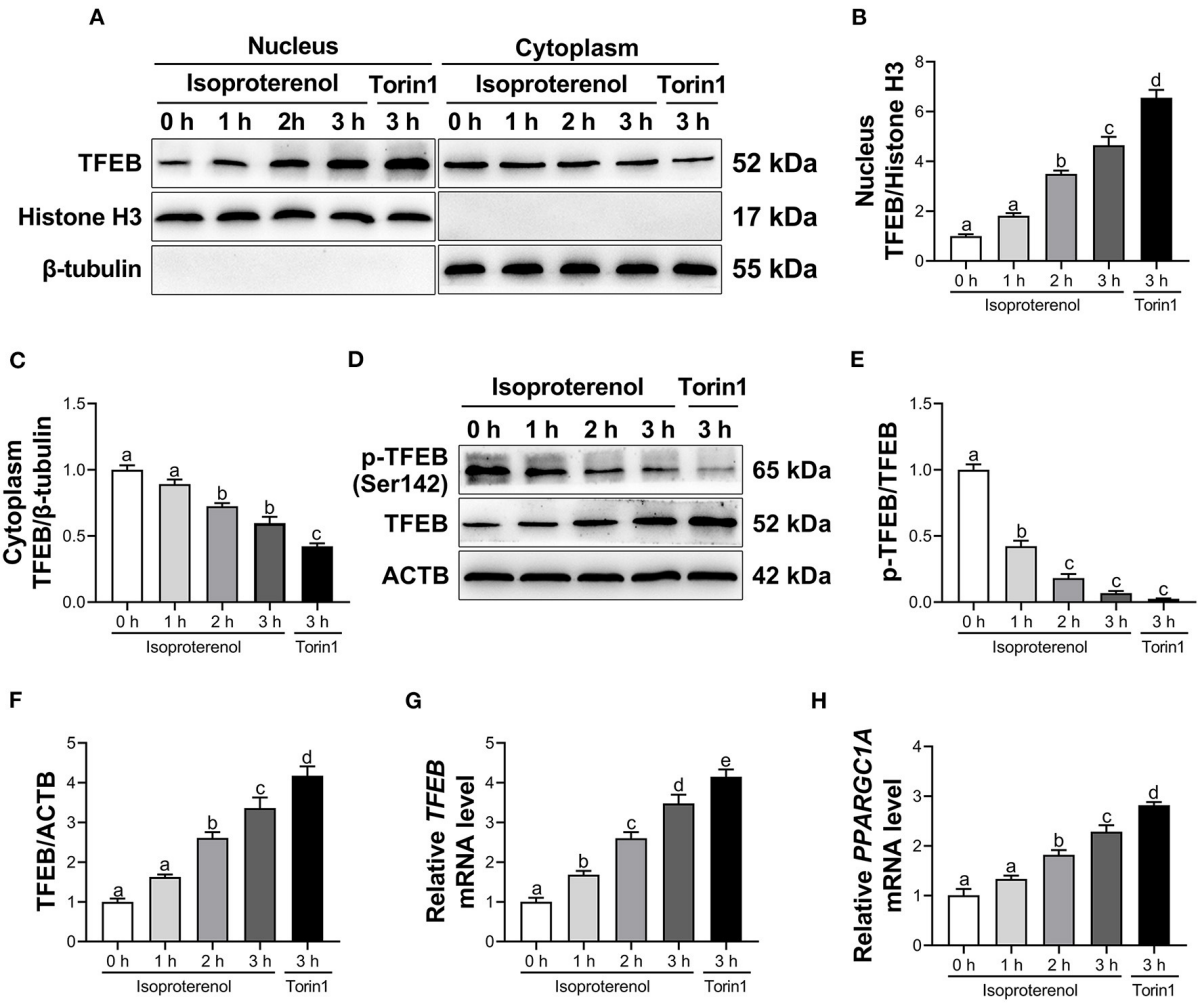
Isoproterenol (ISO) and Torin1 treatment activate transcription factor EB (TFEB) in calf adipocytes. Calf adipocytes were stimulated with 10 μ*M* ISO for 0, 1, 2, or 3 h, or treated with 250 n*M* Torin1 for 3 h. **(A)** Western blot analysis of TFEB in the nucleus and cytoplasm of calf adipocytes. Representative blots are shown. **(B,C)** Quantification of protein abundance of nuclear and cytoplasmic TFEB, respectively. **(D)** Western blot analysis of phosphorylated TFEB (p-TFEB) and total TFEB in calf adipocytes. Representative blots are shown. **(E,F)** Quantification of ratio of p-TFEB/TFEB and protein abundance of TFEB, respectively. **(G,H)** Relative mRNA abundance of *TFEB* and peroxisome proliferator-activated receptor gamma coactivator 1 alpha (*PPARGC1A*) in calf adipocytes. Data were analyzed using one-way ANOVA with Tukey's tests for data meeting homogeneity of variance or with Tamhane's T2 analysis for data of heteroscedasticity and expressed as mean ± SEM. The same letter (a-e) indicates no significant difference (*P* > 0.05), whereas different letters indicate a significant difference (*P* < 0.05).

Compared with the control group, treatment with ISO linearly enhanced nuclear and total protein abundance of TFEB, and linearly reduced cytoplasmic protein abundance of TFEB and ratio of p-TFEB to TFEB (*P* < 0.05, Figures 2A–F and Supplementary Table 2) in adipocytes. Furthermore, there was a quadratic effect on the ratio of p-TFEB to TFEB (Supplementary Table 2) in response to incubation time with ISO. In addition, there was also a linear increase in mRNA abundance of *TFEB* and *PPARGC1A* (*P* < 0.05, Figures 2G,H and Supplementary Table 3) in adipocytes due time of incubation with ISO.

Compared with the control group, treatment with Torin1, an mTOR-dependent TFEB activator, increased nuclear and total protein abundance of TFEB, while decreased cytoplasmic protein abundance of TFEB and ratio of p-TFEB to TFEB (*P* < 0.05, Figures 2A–F) in adipocytes. Furthermore, mRNA abundance of *TFEB* and *PPARGC1A* was upregulated (*P* < 0.05, Figures 2G,H) in Torin1-treated adipocytes.

### Effects of Isoproterenol and Torin1 on Lipolysis

**Figure 3:**
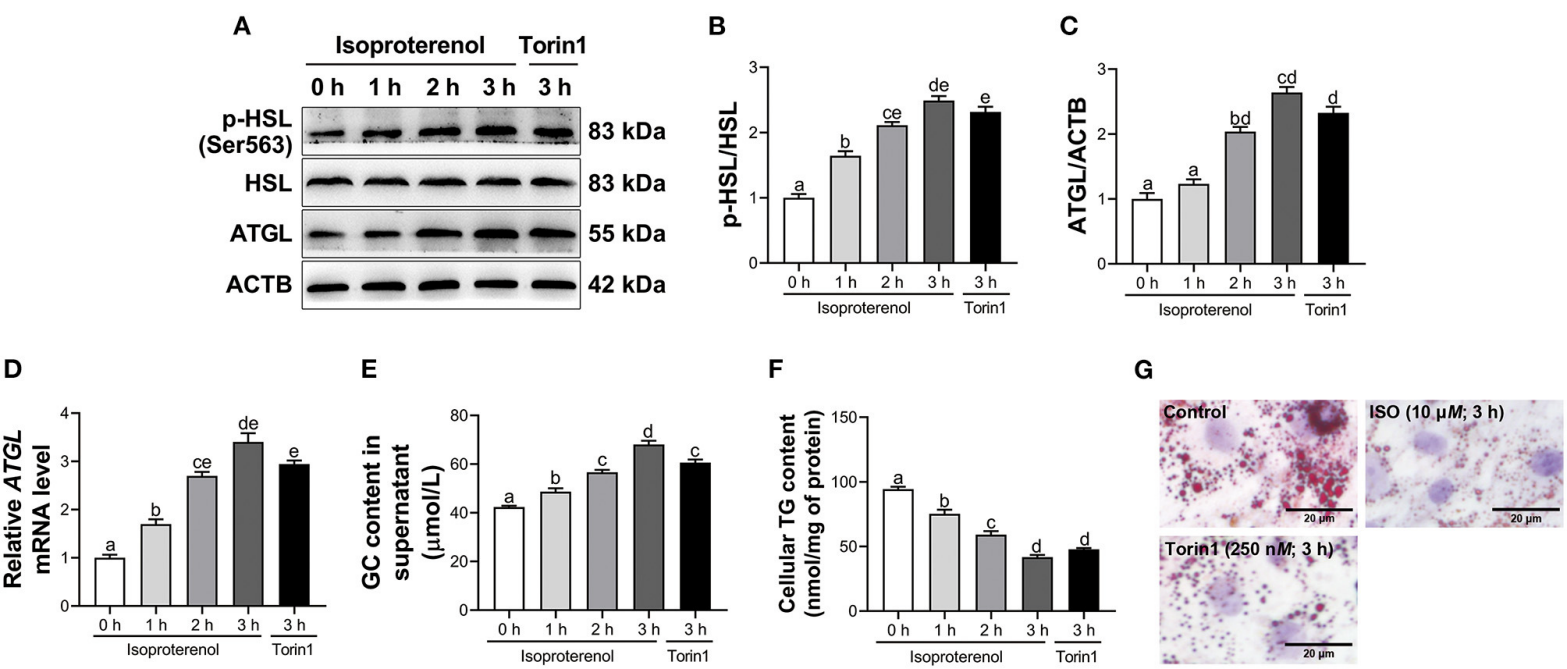
Isoproterenol (ISO) and Torin1 treatment induce lipolysis in calf adipocytes. Calf adipocytes were stimulated with 10 μ*M* ISO for 0, 1, 2, or 3 h, or treated with 250 n*M* Torin1 for 3 h. **(A)** Western blot analysis of phosphorylated hormone sensitive lipase (p-HSL), HSL and adipose triacylglycerol lipase (ATGL) in calf adipocytes. Representative blots are shown. **(B,C)** Quantification of ratio of p-HSL/HSL and protein abundance of ATGL, respectively. **(D)** Relative mRNA abundance of *ATGL* in calf adipocytes. **(E)** The content of glycerol (GC) in the supernatant of calf adipocytes. **(F)** The triglyceride (TG) content in calf adipocytes. **(G)** Oil Red O staining. Scale bar = 20 μm. Data were analyzed using one-way ANOVA with Tukey's tests for data meeting homogeneity of variance or with Tamhane's T2 analysis for data of heteroscedasticity and expressed as mean ± SEM. The same letter (a-e) indicates no significant difference (*P* > 0.05), whereas different letters indicate a significant difference (*P* < 0.05).

A linear increase and quadratic effect on the ratio of p-HSL to HSL were observed with increasing incubation time with ISO (*P* < 0.05, Figures 3A,B and Supplementary Table 4). Furthermore, ISO treatment linearly increased mRNA and protein abundance of ATGL in adipocytes and had a quadratic effect on ATGL protein abundance (*P* < 0.05, Figures 3A,C,D and Supplementary Table 4). Similarly, GC content in supernatant of calf adipocytes increased (*P* < 0.05, Figure 3E and Supplementary Table 5) in a linear and quadratic fashion as a function of incubation time with ISO. In contrast, ISO treatment linearly reduced TG content (*P* < 0.05, Figure 3F and Supplementary Table 5) in adipocytes. Furthermore, Oil Red O staining also showed the same result that ISO decreased the number and size of lipid droplets in adipocytes (Figure 3G).

Compared with the control, treatment with Torin1 increased the ratio of p-HSL to HSL (*P* < 0.05, Figures 3A,B) in adipocytes. In addition, mRNA and protein abundance of ATGL were upregulated (*P* < 0.05, Figures 3A,C,D) in Torin1-treated adipocytes. Similarly, GC content in supernatant of adipocytes treated with Torin1 was greater (*P* < 0.05, Figure 3E). In contrast, TG content was lower (*P* < 0.05, Figure 3F) in Torin1-treated adipocytes. Furthermore, Oil Red O staining also showed that Torin1 treatment decreased the number and size of lipid droplets in adipocytes (Figure 3G).

### Effects of Knockdown of Transcription Factor EB on Lipolysis

**Figure 4:**
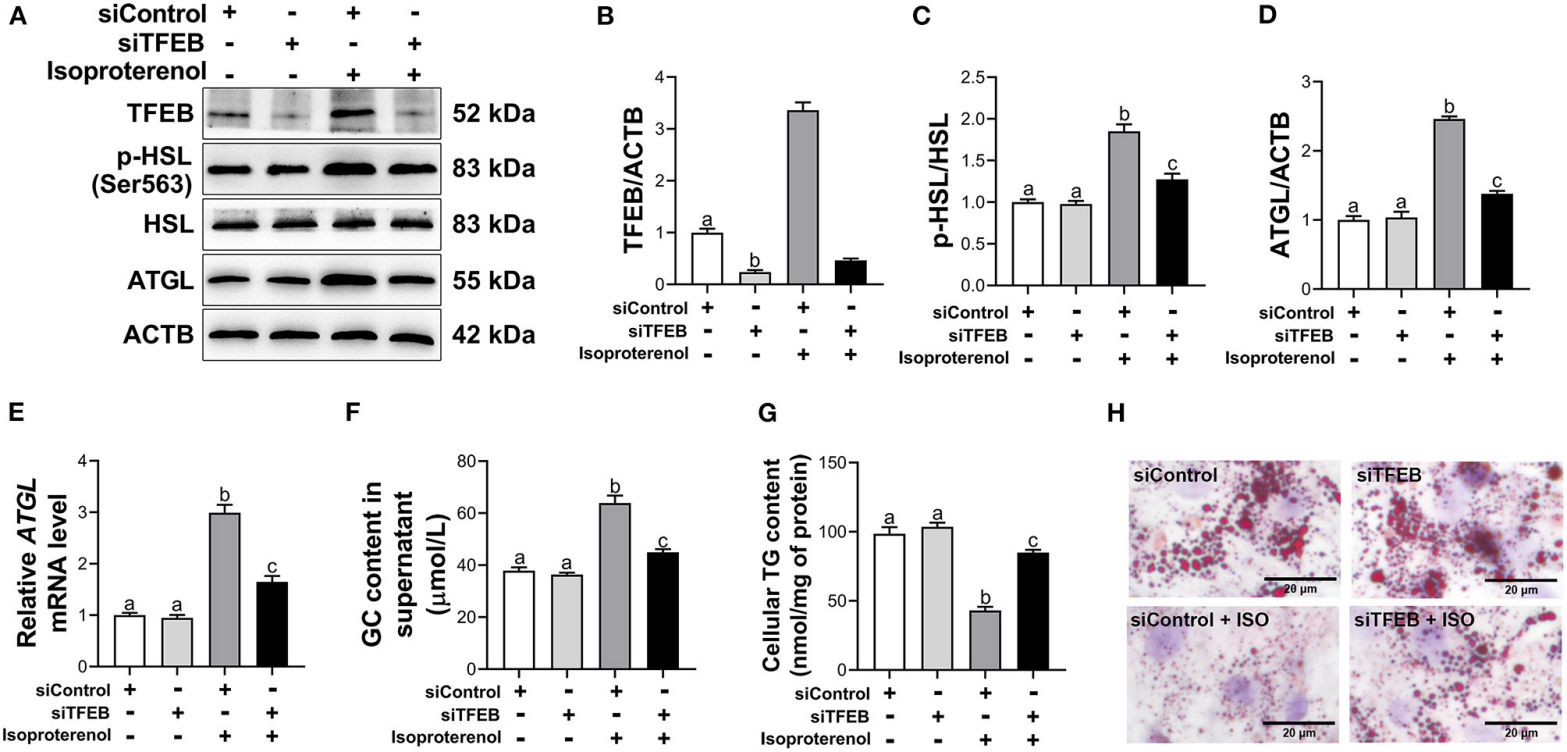
Knockdown of transcription factor EB (*TFEB*) reduces isoproterenol (ISO)-induced lipolysis in calf adipocytes. The cells were divided into 4 groups as followed: an siControl group [transfected with negative control of small interfering RNA (siRNA) for 48 h], and an siTFEB group (transfected with siRNA for TFEB for 48 h), an siControl + ISO group (transfected with negative control of siRNA for 48 h and then treated with 10 μ*M* ISO for 3 h), and an siTFEB + ISO group (transfected with siRNA for TFEB for 48 h and then treated with 10 μ*M* ISO for 3 h). **(A)** Western blot analysis of TFEB, phosphorylated hormone sensitive lipase (p-HSL), HSL and adipose triacylglycerol lipase (ATGL) in bovine adipocytes. Representative blots are shown. **(B–D)** Quantification of protein abundance of TFEB, ratio of p-HSL/HSL and protein abundance of ATGL, respectively. **(E)** Relative mRNA abundance of *ATGL* in calf adipocytes. **(F)** The content of glycerol (GC) in the supernatant of calf adipocytes. **(G)** The triglyceride (TG) content in calf adipocytes. **(H)** Oil Red O staining. Scale bar = 20 μm. Data were analyzed using one-way or two-way ANOVA with Tukey's tests for data meeting homogeneity of variance or with Tamhane's T2 analysis for data of heteroscedasticity and expressed as mean ± SEM. The same letter (a-c) indicates no significant difference (*P* > 0.05), whereas different letters indicate a significant difference (*P* < 0.05).

Transfection with siTFEB downregulated protein abundance of TFEB (*P* < 0.05, Figures 4A,B). Under basal conditions, knockdown of *TFEB* had no significant effect on the ratio of p-HSL to HSL, mRNA and protein abundance of ATGL, GC content in supernatant and TG content in adipocytes (*P* > 0.05, Figures 4A,C–G). Similarly, as assessed by Oil Red O staining of adipocytes, knockdown of *TFEB* had no significant effect on lipid accumulation and lipid droplet size (Figure 4H) under basal lipolysis. However, under ISO-stimulated lipolysis, knockdown of *TFEB* reduced the ratio of p-HSL to HSL (*P* < 0.05, Figures 4A,C). In addition, mRNA and protein abundance of ATGL were downregulated (*P* < 0.05, Figures 4A,D,E) in ISO-treated adipocytes after *TFEB* knockdown. Knockdown of *TFEB* reduced GC content (*P* < 0.05, Figure 4F) in supernatant of ISO-treated adipocytes. In contrast, TG content was greater (*P* < 0.05, Figure 4G) in ISO-treated adipocytes after *TFEB* knockdown. Furthermore, Oil red O staining revealed that knockdown of *TFEB* weakened the effect of ISO on lipid droplets in adipocytes. There were more and larger LDs (Figure 4H) in ISO-treated adipocytes after *TFEB* knockdown.

## Discussion

Ketosis is a common metabolic disorder in high yielding dairy cows as result of NEB-induced fat mobilization (8). Although some studies have evaluated the effects of TFEB on lipid metabolism in murine hepatocytes, little is known about the association between TFEB and lipolysis in ketotic cows (17, 32). In this study, transcriptional activity of TFEB and lipolysis were enhanced in WAT of dairy cows with SCK. Furthermore, activation of TFEB enhanced lipolysis in adipocytes, whereas knockdown of *TFEB* weakened ISO-induced lipolysis. Thus, TFEB may play a crucial role in controlling TG catabolism in adipocytes during a disorder such as SCK.

Lipid mobilization in early lactation dairy cows includes lipogenesis and lipolysis. In this study, the result that mRNA abundance of *ACC1*, a rate-limiting enzyme for fatty acid synthesis (33), and *DGAT1*, involved in TG synthesis (28), did not change significantly in adipose tissue of dairy cows with SCK suggested that lipogenic activity was negligible in early lactation (34). Sustained lipolysis, as an adaptive response to energy deficiency, is a major characteristic of ketotic cows (35, 36). The enzymes ATGL and HSL are rate-limiting in the regulation of TG hydrolysis in adipocytes (37, 38). ATGL performs the first step of TG hydrolysis to produce diglyceride (DG) and FFA (39). Subsequently, DG is converted to monoacylglycerol and FFA by the action of HSL (40). Schoiswohl et al. reported reduced lipolytic activity in adipocytes from *ATGL* knockout mice (41). Furthermore, the phosphorylation site of HSL at Ser residue 563 (Ser563) was found to be important for the lipolytic response of bovine adipose tissue (42). Thus, the upregulated mRNA and protein abundance of ATGL and ratio of p-HSL (Ser563)/HSL ratio in cows with SCK confirmed the importance of these enzymes in the control of lipolytic responses in bovine adipose tissue.

Upon dephosphorylation, TFEB translocates into the nucleus to execute its function as a transcription factor (43). Variants of TFEB carrying Ser-to-Ala mutations of Ser142 is always nuclear localization and constitutively active (13, 44, 45). Thus, decreased phosphorylation of TFEB at Ser142 and increased TFEB nuclear translocation indicated enhanced transcriptional activity of TFEB in adipose tissue of dairy cows with SCK. It has been reported that TFEB can activate its own transcription through a positive feedback loop (10). Furthermore, TFEB regulates *PPARGC1A* abundance by directly binding to the coordinated lysosomal expression and regulation site of the *PPARGC1A* promoter (10). In addition, Evans et al. reported that adipocyte-specific *TFEB* overexpression markedly upregulated *PPARGC1A* abundance in mice fed with HFD (17). Thus, greater mRNA abundance of *TFEB* and *PPARGC1A* in adipose tissue of dairy cows with SCK further underscored increased TFEB transcriptional activity.

Previous studies demonstrated that activated TFEB contributed to lipid catabolism in mouse hepatocytes (32, 46). Consistent with these studies, increased TFEB transcriptional activity and lipolytic response were observed in adipose tissue of cows with SCK, suggesting an association between TFEB and lipolysis in adipocytes. ISO, a major non-selective β-adrenergic receptor agonist, induces lipolysis in bovine adipocytes and differentiated mouse embryo fibroblast (3T3-L1) adipocytes (28, 47). Thus, the fact that ISO treatment increased GC content in supernatant, enzymatic activities of ATGL and HSL and transcriptional activity of TFEB (as demonstrated by increased TFEB nuclear translocation and reduced phosphorylation at Ser142) provided additional evidence for a biological role of TFEB. This was further strengthened by the fact that the TFEB activator Torin1 promoted lipolysis, whereas knockdown of *TFEB* dampened the ISO-induced lipolytic response. In line with our observations, Li et al. reported that the small-molecule compound HEP14 accelerated the degradation of lipid droplets (LDs) by activating TFEB in human HepG2 cells (48). Similarly, overexpression of *TFEB* reduced lipid accumulation in adipose tissues and liver of HFD mice (10, 17, 49). Thus, together, the present results combined with previous studies suggested that activated TFEB may partly induce fat mobilization in dairy cows with SCK.

In the present study, increased TFEB transcriptional activity and lipolysis were observed in adipose tissues of dairy cows with SCK. It is well-accepted that energy deficient leads to lipid mobilization and contributes the occurrence of ketosis. Previous studies also demonstrated that nutritional deprivation increased TFEB transcriptional activity in mouse embryonic fibroblasts and HeLa cells (22, 50). At least in non-ruminants, a clear link exists between TFEB activation and elevated autophagy-lysosomal function (13, 19, 42). Moreover, the autophagic degradation of lipid droplets, termed lipophagy, is a major mechanism that contributes to lipolysis (51). Additionally, it has been found that starvation induced lipid decomposition by activating TFEB-mediated lipophagy in mouse livers and *C. elegans* (10, 21). Thus, it could be possible that NEB-activated TFEB promotes lipophagy in adipose tissues of dairy cows with ketosis.

It is worth to note that knockdown of *TFEB* had no effects on basal lipolysis in adipocytes, which is consistent with the study of Settembre et al. who reported that liver-specific knockout of *TFEB* did not affect the abundances of lipid catabolism genes in the liver of mice under fed conditions (10). Interestingly, transcription factor E3 (TFE3), also a member of Mit/TFE family, regulates a similar set of genes and, similar to TFEB, undergoes cytoplasm-to-nucleus shuttling in response to starvation (48, 52, 53). Pastore et al. reported that overexpression of *TFE3* rescued HFD-induced hepatic steatosis in *TFEB* liver-specific knockout mice, indicating that TFE3 can compensate for TFEB deficiency (49). Thus, under basal condition, TFE3 might compensate for the loss of TFEB and subsequently dampen the effects of knockdown of *TFEB* on lipolysis in adipocytes.

## Conclusions

The lipolytic response and TFEB transcriptional activity were enhanced in WAT of dairy cows with SCK. TFEB activation increased lipolysis, whereas *TFEB* knockdown mitigated ISO-induced lipolysis in calf adipocytes. Accordingly, our results suggested that intense lipolysis was associated with abnormally activated TFEB in WAT of dairy cows with SCK. These findings provide data in support of TFEB as a target in the control of prolonged lipolysis to protect dairy cows from ketosis.

## Data Availability Statement

The raw data supporting the conclusions of this article are available on request to the corresponding author.

## Ethics Statement

The animal study was reviewed and approved by Animals Care and Ethics Committee at Jilin University.

## Author Contributions

HY carried out the experiment, performed the statistical analysis, and wrote original draft preparation. XG, JL, QJ, ZF, XH, ZS, MF, MC, XWL, GL, and ZW contributed in investigation, validation, and animal management. XBL and XD designed the experiment, contributed in conceptualization, formal analysis, and revision of manuscript. All authors contributed to the article and approved the submitted version.

## Funding

This research was funded by the Jilin Province Science and Technology Development Project (Changchun, China; Grant No. 20210508011RQ) and the National Natural Science Foundation of China (Beijing, China; Grant Nos. 32002349 and 32060818).

## Conflict of Interest

The authors declare that the research was conducted in the absence of any commercial or financial relationships that could be construed as a potential conflict of interest.

## Publisher's Note

All claims expressed in this article are solely those of the authors and do not necessarily represent those of their affiliated organizations, or those of the publisher, the editors and the reviewers. Any product that may be evaluated in this article, or claim that may be made by its manufacturer, is not guaranteed or endorsed by the publisher.
