# Supplementary material for: Activation of Transcription Factor EB Is Associated With Adipose Tissue Lipolysis in Dairy Cows With Subclinical Ketosis
Source: Front Vet Sci. 2022 Feb 8;9:816064. doi: 10.3389/fvets.2022.816064 (PMC8861084; doi:10.3389/fvets.2022.816064)
Supplement: Supplementary file 1 [file Data_Sheet_1.docx]

Supplementary Material

# Supplementary Table 1 List of forward and reverse primers used in quantitative real-time PCR analysis.

| Gene | Primer set (5′–3′) | Length (bp) | Accession number |
| --- | --- | --- | --- |
| *TFEB* | For TGCTGACCCCAGATCCAACT | 76 | NM_001205666.1 |
|  | Rev CCCAAACCTGCTTGATCACC |  |  |
| *PPARGC1A* | For CCCGTGCTACCTGAGAGAGA | 125 | NM_177945.3 |
|  | Rev CTTGACTGGGATGACCGAAG |  |  |
| *ACC1* | For TCCTGCTGCTATTGCTACTCCA | 95 | NM_174224.2 |
|  | Rev CAGTCCCCGCACTCACATAA |  |  |
| *DGAT1* | For CCACTGGGACCTGAGGTGTC | 101 | NM_174693.2 |
|  | Rev GCATCACCACACACCAATTCA |  |  |
| *ATGL* | For ACGTGGAACATCTCGTTCGC | 198 | NM_001046005.2 |
|  | Rev CACCTCGATGATGTTGGCAC |  |  |
| *ACTB* | For GCCCTGAGGCTCTCTTCCA | 101 | NM_173979.3 |
|  | Rev GCGGATGTCGACGTCACA |  |  |
| *GAPDH* | For GGCGTGAACCACGAGAAGTATAA | 118 | NM_001034034.2 |
|  | Rev CCTCCACGATGCCAAAGTG |  |  |

# For = forward; Rev = reverse; *TFEB* = transcription factor EB; *PPARGC1A* = peroxisome proliferator-activated receptor gamma coactivator 1 alpha; *ACC1* = acetyl-CoA carboxylase 1; *DGAT1* = diacylglycerol acyltransferase 1; *ATGL* = adipose triacylglycerol lipase; *ACTB* = β-actin; *GAPDH* = glyceraldehyde-3-phosphate dehydrogenase.

# Supplementary Table S2. Effects of different times of ISO treatment (0, 1, 2 or 3 h) on the abundance of proteins associated with transcriptional activity of TFEB in calf adipocytes^1^.

|  | ISO treatment (h) | | | |  | *P*-value | |
| --- | --- | --- | --- | --- | --- | --- | --- |
| Index | 0 | 1 | 2 | 3 | SEM | Linear | Quadratic |
| Nuclear TFEB | 1.00^a^ | 1.82^a^ | 3.50^b^ | 4.65^c^ | 0.34 | < 0.05 | 0.403 |
| Cytoplasmic TFEB | 1.00^a^ | 0.89^a^ | 0.72^b^ | 0.60^b^ | 0.04 | < 0.05 | 0.802 |
| p-TFEB/TFEB | 1.00^a^ | 0.42^b^ | 0.18^c^ | 0.07^c^ | 0.08 | < 0.05 | < 0.05 |
| Total TFEB | 1.00^a^ | 1.63^a^ | 2.61^b^ | 3.36^c^ | 0.22 | < 0.05 | 0.697 |

^1^ Data are expressed as the mean ± SEM. Linear and quadratic contrast were conducted to evaluated time-dependent effects. Comparisons among groups were analyzed using one-way ANOVA followed by Tukey’s tests.

^a-d^ Different superscript letters appearing in the same row indicate a significant difference (*P* < 0.05), whereas the same letter indicates no significant difference (*P* > 0.05).

ISO = isoproterenol; TFEB = transcription factor EB; p-TFEB = phosphorylated TFEB.

# Supplementary Table S3. Effects of different times of ISO treatment (0, 1, 2 or 3 h) on *TFEB* and *PPARGC1A* mRNA abundance in calf adipocytes^1^.

|  | ISO treatment (h) | | | |  | *P*-value | |
| --- | --- | --- | --- | --- | --- | --- | --- |
| Index | 0 | 1 | 2 | 3 | SEM | Linear | Quadratic |
| *TFEB* | 1.00^a^ | 1.69^b^ | 2.60^c^ | 3.48^d^ | 0.23 | < 0.05 | 0.516 |
| *PPARGC1A* | 1.00^a^ | 1.34^a^ | 1.82^b^ | 2.29^c^ | 0.12 | < 0.05 | 0.528 |

^1^ Data are expressed as the mean ± SEM. Linear and quadratic contrast were conducted to evaluated time-dependent effects. Comparisons among groups were analyzed using one-way ANOVA followed by Tukey’s tests.

^a-d^ Different superscript letters appearing in the same row indicate a significant difference (*P* < 0.05), whereas the same letter indicates no significant difference (*P* > 0.05).

ISO = isoproterenol; *TFEB* = transcription factor EB; *PPARGC1A* = peroxisome proliferator-activated receptor gamma coactivator 1 alpha.

# Supplementary Table S4. Effects of different times of ISO treatment (0, 1, 2 or 3 h) on the abundance of genes and proteins associated with lipolytic activity in calf adipocytes^1^.

|  | ISO treatment (h) | | | |  | *P*-value | |
| --- | --- | --- | --- | --- | --- | --- | --- |
| Index | 0 | 1 | 2 | 3 | SEM | Linear | Quadratic |
| p-HSL/HSL | 1.00^a^ | 1.64^b^ | 2.11^c^ | 2.49^d^ | 0.13 | < 0.05 | < 0.05 |
| *ATGL* | 1.00^a^ | 1.70^a^ | 2.70^b^ | 3.40^c^ | 0.22 | < 0.05 | 0.991 |
| ATGL | 1.00^a^ | 1.23^a^ | 2.04^b^ | 2.64^c^ | 0.15 | < 0.05 | < 0.05 |

^1^ Data are expressed as the mean ± SEM. Linear and quadratic contrast were conducted to evaluated time-dependent effects. Comparisons among groups were analyzed using one-way ANOVA followed by Tukey’s tests.

^a-d^ Different superscript letters appearing in the same row indicate a significant difference (*P* < 0.05), whereas the same letter indicates no significant difference (*P* > 0.05).

ISO = isoproterenol; p-HSL = phosphorylated hormone sensitive lipase; *ATGL* = adipose triacylglycerol lipase.

# Supplementary Table S5. Effects of different times of ISO treatment (0, 1, 2 or 3 h) on GC content in supernatant and TG content in calf adipocytes^1^.

|  | ISO treatment (h) | | | |  | *P*-value | |
| --- | --- | --- | --- | --- | --- | --- | --- |
| Index | 0 | 1 | 2 | 3 | SEM | Linear | Quadratic |
| GC (μmol/L) | 42.38^a^ | 48.70^b^ | 56.62^c^ | 68.08^d^ | 2.27 | < 0.05 | < 0.05 |
| TG (nmol/mg) | 94.33^a^ | 75.29^b^ | 59.09^c^ | 41.86^d^ | 4.59 | < 0.05 | 0.714 |

^1^ Data are expressed as the mean ± SEM. Linear and quadratic contrast were conducted to evaluated time-dependent effects. Comparisons among groups were analyzed using one-way ANOVA followed by Tukey’s tests.

^a-d^ Different superscript letters appearing in the same row indicate a significant difference (*P* < 0.05), whereas the same letter indicates no significant difference (*P* > 0.05).

ISO = isoproterenol; GC = glycerol; TG = triglyceride.

# Supplementary Figure S1

#
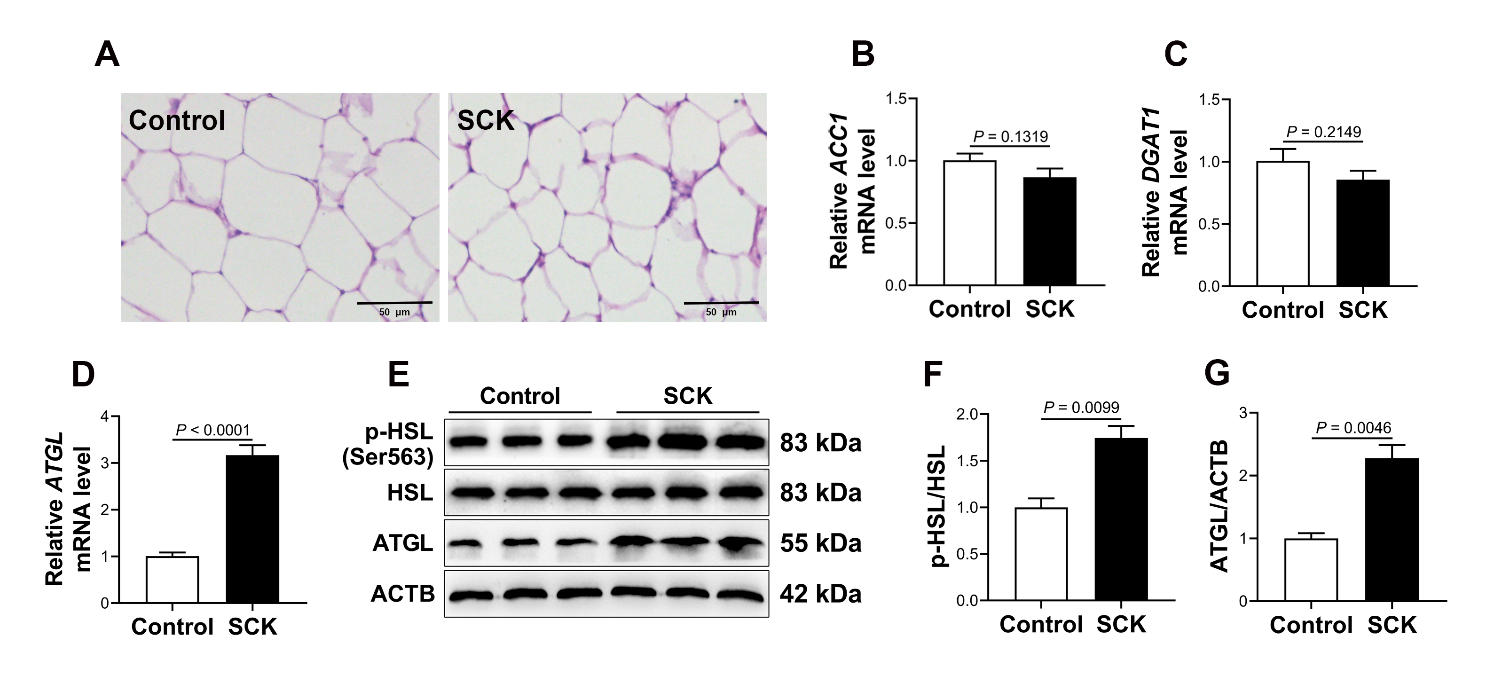


# Supplementary Figure S1. Lipolysis status in white adipose tissue (WAT). (A) Morphologic changes in WAT of control cows (n = 15) and dairy cows with subclinical ketosis (SCK; n = 15) investigated following hematoxylin and eosin (HE) staining. Scale bar = 50 μm. (B-D) Relative mRNA abundance of acetyl-CoA carboxylase 1 (*ACC1*), diacylglycerol acyltransferase 1 (*DGAT1*), and adipose triacylglycerol lipase (*ATGL*) in WAT of control cows (n = 15) and dairy cows with SCK (n = 15). (E) Western blot analysis of phosphorylated hormone sensitive lipase (p-HSL), HSL, and ATGL in WAT of control cows (n = 15) and dairy cows with SCK (n = 15). Representative blots are shown. (F, G) Quantification of ratio of p-HSL/HSL and protein abundance of ATGL, respectively. Data were analyzed using unpaired *t*-tests and expressed as mean ± SEM.
